# Recent advances in the molecular epidemiology of clinical malaria

**DOI:** 10.12688/f1000research.14991.1

**Published:** 2018-07-30

**Authors:** Mario Recker, Peter C Bull, Caroline O Buckee

**Affiliations:** 1 Centre for Mathematics and the Environment, University of Exeter, Penryn Campus, Penryn, TR10 9FE, UK; 2 Department of Pathology, University of Cambridge, Cambridge, CB2 1QP, UK; 3 Center for Communicable Disease Dynamics, Harvard T.H. Chan School of Public Health, Boston, MA, 02115, USA

**Keywords:** malaria, epidemiology, severe disease, natural acquired immunity, infectivity

## Abstract

Human malaria is a complex disease that can show a wide array of clinical outcomes, from asymptomatic carriage and chronic infection to acute disease presenting various life-threatening pathologies. The specific outcome of an infection is believed to be determined by a multifactorial interplay between the host and the parasite but with a general trend toward disease attenuation with increasing prior exposure. Therefore, the main burden of malaria in a population can be understood as a function of transmission intensity, which itself is intricately linked to the prevalence of infected hosts and mosquito vectors, the distribution of infection outcomes, and the parasite population diversity. Predicting the long-term impact of malaria intervention measures therefore requires an in-depth understanding of how the parasite causes disease, how this relates to previous exposures, and how different infection pathologies contribute to parasite transmission. Here, we provide a brief overview of recent advances in the molecular epidemiology of clinical malaria and how these might prove to be influential in our fight against this important disease.

## Background

Malaria is caused by the mosquito-transmitted protozoan parasite *Plasmodium spp*. There are five species known to infect humans: *P. falciparum*, *P. vivax*, *P. malariae*, *P. ovale*, and *P. knowlesi*, of which *P. falciparum* malaria is the most virulent form, causing the most morbidity and mortality in humans, and thus will be the focus here. Despite continual control efforts over the last 50 years or more, the burden of disease is still substantial, and recent estimates put the annual number of malaria cases in the region of more than 200 million, leading to over 400,000 deaths, predominantly in young children living in sub-Saharan Africa ^1^. The first licensed malaria vaccine (RTS,S) has shown limited efficacy ^2^ and currently is introduced as only a pilot scheme in a few settings in sub-Saharan Africa. The main disease intervention therefore still relies on drug treatment of patients and prevention of exposure by (insecticide-treated) bed nets, spraying of houses with insecticides, and other general mosquito control measures. Following a large scale-up of vector control in combination with artemisinin combination therapy, there has been an overall reduction in the number of malaria cases over the last decade ^3^. However, this reduction does not always correspond well with known control measures ^4^ and shows significant geographic variations. For example, big reductions have been achieved in Southeast Asia and the Western Pacific, whereas several regions in the Americas and Africa have experienced no change or even an increase in cases in recent years ^1, 5^.

The challenges in malaria control are manifold, and, even in regions where a drastic reduction has been achieved and where local elimination is theoretically possible, maintaining a disease-free state without achieving a similar reduction in neighboring regions will be difficult. Underlying these challenges is a lack of understanding of the basic biology of malaria transmission and its relationship to the epidemiological patterns of infection and disease in different transmission settings. In particular, not all infections cause severe clinical symptoms, and most infections contributing to transmission in a given location cause only mild illness or are classified as asymptomatic (but see 6 for a critical discussion on the terminology of “asymptomatic” malaria). Intervention-induced changes in parasite transmission therefore will incur shifts in the age distributions of particular age- and exposure-dependent disease manifestations in complex ways ^7, 8^. Predicting the epidemiological outcome of control measures therefore requires a more in-depth knowledge of the factors responsible for severe malaria as well as a better understanding of who is currently infected and who contributes to transmission.

## What causes severe malaria?

Malaria infections are initiated by the bite of an infectious mosquito, which releases sporozoites into the bloodstream that subsequently travel to, and undergo differentiation into merozoites in, the liver. After multiple rounds of multiplication within infected liver cells, merozoites are released into the bloodstream, starting a cycle of repeated invasion and multiplication within red blood cells (RBCs) that leads, both directly and indirectly, to considerable cell destruction. In addition to anemia as a direct result of RBC loss, splenic clearance of uninfected RBCs, and reduced RBC production, malaria pathology is often caused by parasites sequestering in the deep vasculature, leading to local inflammation, hemorrhages, tissue damage, and obstruction of blood flow. Sequestration itself is the result of infected RBCs (iRBCs) adhering to a number of different host endothelial cell receptors ^9– 12^ through highly polymorphic parasite proteins called PfEMP1 ^13^ that are encoded by the *var* multigene family ^14^ and inserted into the surface of iRBCs. The prominent expression of these proteins on the surface of iRBCs makes them key targets for adaptive immune responses, which the parasite escapes by exploiting the enormous sequence variation of *var* genes both between multiple variant *var* gene copies within individual parasites and between repertoires of *var* genes within parasite populations. In one of the most sophisticated immune-evasion strategies studied, the parasite can switch between different PfEMP1 types during infection in a process referred to as clonal antigenic variation ^15, 16^.

Despite its diversity, PfEMP1 plays a central role as a target of naturally acquired immunity (NAI). Over years of repeated infections, individuals living in malaria-endemic areas acquire a repertoire of PfEMP1 variant-specific immune responses through repeated infections that are believed to confer protection from life-threatening disease (reviewed in 17). In a clear illustration of the importance of PfEMP1 as immune targets, women in their first pregnancy who have grown up in malaria-endemic areas and who have gained immunity to severe malaria temporarily lose this immunity because their placentas open up a novel niche for parasite sequestration. This is exploited by a single functionally and immunologically distinct PfEMP1 type, VAR2CSA (see below), which is present in every parasite genome and to which immunity is rapidly gained ^18^.

So far, the high diversity of PfEMP1 has precluded this family of molecules from being considered a serious vaccine target. However, the discovery that certain disease manifestations are associated with the expression of restricted subsets of PfEMP1 variants has opened up the debate of whether an anti-disease or anti-virulence vaccine in fact might be a feasible option ^19^. One of the first and so far most robust examples is the involvement of a particular PfEMP1 variant in pregnancy-associated malaria, mediated by the binding of VAR2CSA-expressing iRBCs to placental chondroitin sulfate A (CSA) ^20^. The fact that this protein appears unusually conserved is now being exploited in the design of the first placental malaria vaccines that are currently undergoing clinical tests ^21, 22^.

This functional subdivision of *var* genes can be extended to those that are involved in childhood malaria. For example, based on upstream promoter sequence (Ups), *var* genes can be divided into three groups—UpsA, UpsB, and UpsC—of which UpsA genes are frequently found to be upregulated during severe infection, particularly in young children. Although sequence diversification within this UpsA group of genes appears to be more restricted than others, they are still too diverse as a whole to be considered potential vaccine targets. As such, the recent discovery that a much smaller gene subset, those containing specific domain types called CIDRα1, and their binding to the endothelial protein C receptor (EPCR) appeared to be associated with cerebral malaria caused great excitement ^23– 26^. In fact, confidence in the importance of this interaction underlying severe infection outcomes is such that it is now being promoted as a potential anti-disease vaccine target ^19^.

However, the crux of the problem is that, in most cases, findings are based on observed associations between pathological outcomes and the proportional expression of gene variants within the infecting parasite population sampled from peripheral blood rather than directly from parasites that are sequestered in tissues. The issues with these kinds of studies are further compounded by the enormous technical challenge of fully taking into account individuals’ exposure histories and therefore their immune status at the time of infection. This means that caution has to be exercised when trying to infer causality, which was recently re-emphasized by Azasi *et al*., who found that *in vitro* iRBC binding to endothelial cells is often independent of EPCR and can easily be interrupted under flow conditions ^27^. Approaches to gain a better understanding of the host–parasite interaction in capillaries include (1) improved understanding of the parasites actually responsible for pathology through direct sampling of sequestered parasites in different tissues by either using skin biopsies in patients ^28^ or sampling from tissues post-mortem ^29^; (2) improved assessment of parasite sequestration in tissues through direct observation of parasites within capillaries through mucosal surfaces to correlate capillary congestion with disease outcome ^30^; (3) seeking associations between peripheral parasite gene expression levels and direct measures of sequestration through malarial retinopathy ^25, 31^; and (4) improved understanding of the role of NAI in shaping the infecting parasite population by seeking associations between peripheral parasite gene expression levels and pre-existing antibody responses in controlled human infections of volunteers with differing levels of natural exposure to infection ^32^.

## Who is infected and who contributes to transmission?

As the outcome of an infection is partially determined by an individual’s exposure history to the parasite, elucidating the clinical epidemiology of malaria requires an understanding of a region’s (spatially and time-varying) transmission intensity and therefore knowledge of who is currently infected, who contributes to transmission, and how much. However, measuring disease prevalence and relating this prevalence to transmission remain important challenges that can be severely hampered by the relatively high proportion of clinically silent and low-parasite density infections, especially in highly endemic settings. With the improvement of molecular methods for parasite detection, it has become increasingly clear that microscopy—still the gold standard for diagnosis in many places—systematically misses a large number of low-density infections. Microscopy detects parasite densities in the blood of greater than about 100 parasites per microliter, a detection threshold similar to that of rapid diagnostic tests, but misses an average of half of all malaria infections compared with standard polymerase chain reaction (PCR) ^33^. Paradoxically, this appears to be the case regardless of transmission setting or exposure/immunity, and a higher fraction of submicroscopic infections occur in low-transmission settings. Indeed, it has been estimated that in areas with less than 10% prevalence by PCR, 88% of infections would not be detected by microscopy ^33^. Recent ultrasensitive PCR techniques ^34^ have lowered this detection limit even further to 22 parasites per milliliter, which has led to researchers confirming the substantial reservoir of low-density infections. In the absence of molecular methods for routine surveillance, understanding the relationship between clinical cases (which form the basis of surveillance in most endemic countries) and overall prevalence of infection remains a key challenge.

The prevalence of infection is not the only consideration for defining transmission, however. Many infections are composed of multiple parasite clones, and new infections often occur and cause new episodes of disease against the backdrop of ongoing asymptomatic parasitemia. This means that even with accurate estimates of the fraction of infected people, we are still unable to describe the incidence or force of infection, which is related to the number of new infections over time. The enormous genomic diversity of the parasite, coupled with the frequency of low-density infections, makes it difficult to detect how many clones each infection is composed of, and there are few robust strain markers with which to follow chains of transmission. Recently, new sequencing methods and accompanying analytical tools have shed light on the extent of superinfection, revealing substantial “complexity of infection”, particularly in high-transmission settings like Uganda where individuals can harbor up to 20 clones ^35^. One result of these findings is a move toward the concept of using the molecular force of infection (molFOI), which measures the number of new genotypes acquired by individuals over time ^36^, to define transmission settings. An added value of these highly sensitive diagnostic tools is that they allow tracking of the genetic relatedness between parasites or infections and, with it, the identification of transmission chains and focal transmission areas. The latter will be particularly important for regions that are nearing malaria elimination and where the monitoring and characterization of residual transmission will be key for sustained malaria control.

The surveillance issues described above are all designed to measure asexual parasites in the blood, which cause the clinical manifestations of malaria but cannot be transmitted to mosquitoes. This means that quantifying the infectious reservoir requires different approaches. Only a small fraction of blood-stage parasites develop into male and female gametocytes, the sexual parasite stages that are taken up during a blood meal and underlie infectiousness. The molecular pathways responsible for the switch to sexual development and the dynamics of gametocytes in the body, as well as their relationship to transmission, are still mysterious ^37^. Furthermore, early notions that directly relate asexual parasitemia with infectivity, which implied that young individuals suffering from severe disease are by far the highest contributor to malaria transmission, have also been put into question by revealing that asymptomatic infections contribute significantly more to transmission than previously thought ^38– 40^. For example, a recent study in Ethiopia used mosquito blood-feeding experiments to establish that only 15% of *P. falciparum*-infected individuals were infectious and that asexual parasitemia was not correlated with infectiousness ^40^. Earlier findings that gametocytes are not homogeneously distributed within the blood and may cluster under the skin to promote transmission ^41, 42^, plus the considerable uncertainties associated with the determinants of parasite fitness in the mosquito as it transitions from sexual gametocytes to infectious sporozoites, mean that there are still some outstanding difficulties in quantitatively linking standard measures of prevalence with transmission intensity.

## Going forward

The identification of EPCR-binding phenotypes and their potential involvement in cerebral malaria has caused excitement and raised some optimism about the possibility of developing an anti-disease vaccine. However, for a convincing case to be made, one still needs to unambiguously demonstrate the causal link between host receptor binding and specific disease manifestations. A crucial point here is that every parasite contains in its repertoire most, if not all, of these “disease-causing”, or rather disease-associated, variants. That is, if the parasite has the freedom to express its entire PfEMP1 antigenic repertoire during infection, what determines the actual outcome? It has been shown that PfEMP1 expression is hierarchical ^43, 44^ and that host immune responses have an influence on what variants are expressed during infection ^32, 45^. However, this alone cannot explain why an infection causes cerebral malaria in one child but severe malarial anemia in another. Furthermore, the observed hierarchical expression of *var* genes may be due simply to the existence of alternative molecular strategies used by the parasites to evade immune responses in individuals of different levels of immunity. There are reported examples of asymptomatic infections that exhibit high levels of expression of group A-like *var* genes previously found to be associated with severe malaria, which suggests that the PfEMP1 antigens they encode can play a role in the maintenance of chronic infections ^46^. At this point, more integrated (that is, systems and -omics) approaches should be able to offer more detailed information about the specific immunological and parasitological processes involved in the progression toward disease, especially when taking into consideration the composition of the infecting parasite population in relation to the host’s immune history (see 47 and 48 for recent examples).

More advanced approaches are also required for improving our understanding of NAI to malaria. Crucially, this necessitates (1) a clear definition of what constitutes protection and (2) robust and measurable correlates of protection, neither of which are straightforward. As mentioned above, NAI has to be considered as a multi-stage process or even a continuum whereby infection severity generally attenuates with cumulative exposure to infection. Numerous studies have tried to find correlates of protection by means of prospective cohort studies in which individuals’ immune responses to predefined panels of antigens are correlated with the incidence of clinical episodes. One of the main problems with these studies is the often small effect size, leading to contradictory findings and poor reproducibility (reviewed in 49). This is further complicated by the lack of reliable measures of how often an individual has been challenged in the past, which is an essential consideration given that the needle of protective responses and the haystack of non-protective responses, as well as NAI itself, all increase with cumulative exposure to infection. Furthermore, condensing this multifaceted process into a binary phenotype (protected or not) bypasses some of the aforementioned complexities underlying malaria pathology and NAI and thus is unlikely to provide a comprehensive picture of the myriad of processes involved.

In that respect, it is also imperative to embrace more sophisticated methods to analyze increasingly complex datasets. Machine learning approaches offer a number of advantages over more traditional, univariate analyses in their ability to extract non-linear relationships and interactions from high-dimensional data in a hypothesis-free manner. For example, in a recent study, we used a machine learning approach to identify predictive signatures of clinical protection from protein microarray data containing thousands of measured immune markers ^50^. In another study, Helb *et al*. used a predictive framework based on machine learning to estimate recent exposure to the malaria parasite ^51^. However, these powerful methods crucially rely on detailed and robust datasets that permit appropriate cross-validation and verification of research findings. One important step forward in that direction is the use of *ensemble* datasets across a wide range of studies, as was recently advocated in order to better define the infectious reservoir and measure transmission more accurately ^52^.

Finally, a more improved understanding of the biology of mosquito–human and human–mosquito transmission needs to include better knowledge of local vector ecologies. Surprisingly, we still know relatively little about how changes in mosquito abundance and species distributions over the last few years and decades—some of the most important determinants of malaria epidemiology—might have not only influenced but actively shaped some of the observed changes in malaria incidence. Unfortunately, detailed and long-term surveillance data on vector distribution are scarce and are available for only a small number of vector species and epidemiological settings. Therefore, large-scale vector sequencing initiatives, such as the malariaGEN 1000 genomes project ^53^, together with more detailed investigations into the behavioral and ecological factors underlying this part of the transmission cycle, will have a central role to play in developing a fine-grained and holistic understanding of malaria epidemiology that incorporates the *Plasmodium* parasite, the human host, and the mosquito vector.
